# On Methodology and Method in Acmeist Psychology

**DOI:** 10.1007/s12124-021-09634-8

**Published:** 2021-09-13

**Authors:** Carlos Kölbl

**Affiliations:** Department of Educational Sciences, Faculty of Humanities and Social Sciences, University of Bayreuth, Bayreuth, Germany

**Keywords:** Vygotsky studies, Objective-analytical method, Method of double stimulation, Semic method, Acmeist psychology, Russian literature, Semiotics, Psychology and art, Single cases

## Abstract

This article asks what kind of science psychology should be and what new readings of Vygotsky can contribute to answering this question. Methodology and method are key to constituting psychology as a science. Hence, the focus is on three major methodologic-methodic approaches to what Vygotsky referred to in his *Notebooks* towards the end of his life as his and his colleagues’ “acmeist psychology” – the objective-analytical, the method of double stimulation and the semic method. Each will be discussed in its own right, followed by a discussion of the interrelatedness of the three in order to provide stimulation for future possibilities. These possibilities – it will be argued – lie in decisively re-orienting psychology as a science that brings single cases and complex semiotic analyses to the fore and thereby also rethinks psychology’s relation towards the arts, especially literature.

As is well known, one can find a number of different internal and external designations for Vygotsky’s and his colleagues’ approach to psychology. These include “cultural psychology,” “historical psychology,” “instrumental psychology,” “socio-cultural approach,” “cultural-historical psychology” and “height psychology” (in contrast to “depth psychology,” Freudian or otherwise). Interestingly, yet another occurs twice in Vygotsky’s *Notebooks* and as far as I know nowhere else: “acmeist psychology” (on Vygotsky’s *Notebooks*: Kölbl & Métraux, 2021). The first instance is prior to the end of 1932 and alongside the term “height psychology”; the second, in 1933, is related to the claim that Baruch Spinoza’s theory implicitly contained the whole acmeist psychology that Vygotsky and his colleagues would develop explicitly (Vygotsky, 2018, p. 292, 375). “Acmeist” is etymologically derived from the Greek word *ἀκμή*, meaning “peak” or “zenith,” so one might, like the editors of the *Notebooks*, equate acmeist with “height psychology” (ibid., p. 303). There is reason, however, to believe that Vygotsky may have had other connotations in mind as well: around 1912, the Russian Modernist poetic current named Akmeizm emerged, linked to such poets as Nikolai Gumilev, Anna Akhmatova and Osip Mandelstam. The last of these appears in *The Psychology of Art* (Vygotsky, 1971, p. 44) and, with lines from his poem “The Swallow,” in the last chapter of *Thinking and Speech* (Vygotsky, 1987, p. 43); Gumilev is also quoted there (ibid., p. 284). This is no accident: “These quotations from the two great Acmeist poets thus summarized the subject of Vygotsky’s chapter: word and thought presuppose each other, but are distinguishable and interact in exceedingly complex ways, that remind us of the interplay of form and content in literary works.” (Van der Veer & Valsiner, 1991, p. 360) Moreover, Vygotsky owned an autographed copy of Osip Mandelstam’s *Tristia* and was acquainted not only with him but also with his wife Nadezhda. They were in contact in Moscow in 1933, the year in which Osip wrote his poem *The Kremlin Highlander* (also known as the *Stalin Epigram*) and one year before the couple was banished (Vygotsky, 2018, p. 267; Mandelstam, 1989, p. 223). (Did Vygotsky get to know the poem there and at that time? Probably not, since he does not seem to have been among his close friends.) Mandelstam has defined acmeism as “a yearning for world culture” (cf. e.g. Doherty, 1995, p. 243 seq.). Such a yearning was not alien to Vygotsky either. (A comparison between acmeist poetic theory, as well as its more general cultural and philosophical dimensions, on the one hand [cf. ibid., in particular chapters three and five] and Vygotsky’s psychology on the other might yield interesting results but goes beyond the scope of this paper). In any case, Vygotsky pursued wide interests in literature and other forms of art and personally knew not only Mandelstam but also Boris Pasternak, Sergei Prokofiev, and Sergei Eisenstein, among others. Whatever Vygotsky exactly had in mind when he designated his and his colleagues’ approach acmeist, the denomination clearly underlines (once more) his affinity for art in general and literature in particular. Reflecting upon this affinity will be the *basso continuo* of the present paper (for a partly related endeavor cf. Vassilieva & Zavershneva, 2020).

However, for any kind of psychology – acmeist or otherwise – methodology and method are crucial. René van der Veer and Valsiner (1991, p. 141) quote a letter Vygotsky wrote to Luria in 1926 that points exactly in this direction: “For me the primary question is the question of method, that is for me the question of truth....” Hence, the remainder of this paper will focus upon three methodologic-methodic approaches of Vygotsky at different times of his life. This will help answer the main question of the present article: what should psychology as a science look like and what can new readings of Vygotsky contribute to this? While also referring to Vygotsky’s writings – his *Notebooks*, texts published in his *Collected Works* or elsewhere – as well as to secondary literature, I will (necessarily) be extremely selective and cursory. Hopefully, it will nevertheless become evident that the discussion of the three approaches will not only permit insights into problems long passed by or overcome and thus anachronistic but also offer some stimulus for future possibilities of re-orienting scientific psychology.

## The Objective-Analytical Method

Vygotsky introduces the “objective-analytical method” right away in the first chapter of his book *The Psychology of Art* (Vygotsky, 1971, pp. 9–26; in particular 26–27). The subject of this monograph is the “aesthetic reaction” (ibid.). Instead of – as one might expect – focusing on persons doing things like reading novels, watching films, going to exhibitions, or listening to music, Vygotsky analyzes the work of art itself: “For the psychologist any work of art is a system of stimuli, consciously and intentionally organized in such way as to excite an aesthetic reaction. By analyzing the structure of the stimuli we reconstruct the structure of the reaction.” (ibid.) Obviously, Vygotsky advocates a sort of indirect method to analyzing this reaction. Moreover, he approaches his subject via the analysis of a few single cases: his beloved Hamlet, some fables by Ivan Krylov, and the story “Gentle Breathing” by Ivan Bunin. In doing this, he is neither interested in specific aesthetic reactions to Shakespeare’s Hamlet, the fables of Krylov, or the story by Bunin as individual works nor those to fables, works of theater or (short) stories as genres of art but aims at gaining *general* insights into the aesthetic reaction as such. “The method of *The Psychology of Art* is analysis, abstraction (that is why there is not a word about the fable as such; I ignore its specific traits).” (Vygotsky, 2018, p. 76)

We again encounter the method in *The Historical Meaning of the Crisis in Psychology* (Vygotsky, 1997), this time embedded in more general reflections on the philosophy of science and methodology. Soon after rejecting the idea of studying psychology *more geometrico* (what would Jan Smedslund say to this?) Vygotsky turns to the objective-analytical method: “The method of analysis in the natural sciences and in causal psychology consists of the study of a *single* phenomenon, a typical representative of a whole series, and the deduction of a proposition *about the whole series* on the basis of that phenomenon.” (ibid., Vygotsky’s emphasis) This formulation, written in 1926, shows a remarkable similarity to Kurt Lewin’s reflections of 1930–1931 on the “Galilean” versus the “Aristotelian” form of thinking in psychology and to his thoughts on law and experiment of 1927 (Lewin, 1981a, 1981b). Hence, Vygotsky may not only have regarded Lewin as having been an important sparring partner in the semantic field (Vygotsky, 2018, pp. 403–417) but also in the methodologic-methodic during the latter’s short stay in Moscow in 1933. Perhaps Gita Vygodskaja heard them talk about the crisis in psychology and the role of the single case then. Vygotsky continues: “When our Marxists explain the Hegelian principle in Marxist methodology they rightly claim that each thing can be examined as a microcosm, as a universal measure in which the whole big world is reflected. On this basis they say that to study one single thing, one subject, one phenomenon *until the end*, exhaustively, means to know the world in all its connections.” (Vygotsky, 1997, p. 317; Vygotsky’s emphasis) A few pages later, he will claim that *Capital* by Karl Marx follows in principle the same methodological idea (ibid., p. 320 seq.). He offers two examples to illustrate the objective-analytical method (ibid., pp. 318–320): on the one hand, his analyses in *The Psychology of Art* and on the other, perhaps more surprisingly, Pavlov’s studies on the salivary gland. In describing these, Vygotsky highlights the ingenuity of the Russian physiologist in dealing with the single case: “Pavlov maximally abstracted the phenomenon he studied from the specific conditions of the particular phenomenon. He brilliantly *perceived the general in the particular*.” (ibid, p. 318; Vygotsky’s emphasis)

## The Method of Double Stimulation

Vygotsky’s methodologic-methodic thinking does not end with the objective-analytical method. The method of double stimulation used in the investigations on concept formation he conducted with Lev S. Sakharov and also in Leont′ev’s study on memory was inspired by the Würzburg School psychologist Narziß Ach (Vygotsky, 2018, pp. 113–128; Vygotsky, 1987, pp. 121–241). In the end, however, this method may not only have been inspired by Ach but also vanguard literary experiments within the transrational poetry of Russian futurism, as Métraux (2006) has suggested.

Let us first recall the investigations on concept formation. The experimental procedure, using the method of double stimulation, consisted in showing the subject several different objects varying in color, form and dimension. Afterwards, the subject was again shown the bottom part of one of the objects, now with one of a set of meaningless three-character words – “bat,” “dek,” “roc,” or “mup”– that had been assigned according to the specific characteristics of the respective object and asked to gather all objects he or she supposed to share the same word. After each trial, the experimenter corrected the subject and revealed the correct name of the object. Employing this method, Vygotsky concluded that concept development is composed of three basic stages: syncretism, complexive thinking and conceptual thinking. These stages are themselves subdivided into distinct phases. In the first stage (syncretism), objects are subsumed under a concept because of, for example, the principle of trial and error, idiosyncratic criteria of classification or the spatial distribution of the objects. In the second stage (complexive thinking), the classification process becomes more systematic. Now, certain objective features of the figures become criteria for differentiating between the objects. Complexive thinking is subdivided into four phases. For our purposes, one of these is especially important, the one during which classifications are based on pseudo-concepts. Figures are then ordered according to objective criteria which also would be crucial for classifications based on real concepts. Vygotsky’s reason for speaking of a pseudo-concept is that the child still classifies the figures according to visible and concrete features and not – as would be typical with real concepts – according to certain principles of abstract thinking. These differences only become visible when the process of thought that led to the classifications is analyzed. On a “phenotype level,” however, the results of the classification based on a pseudo-concept cannot be distinguished from a classification based on a real concept. They constitute, if you will, functional equivalents and, according to Vygotsky, make communication with adults a lot easier. Only at the very end of the process is the child or adolescent able to make classifications based on real concepts. Or, in other words, “[…] the investigations proved the idea […] that children, adolescents, and adults may mean different things by the same words. It showed that children’s learning of words marks only the onset of a semantic development that may take years to reach its culmination point. In a way, then, children and adults are living in a *different semantic universe* and the words they use coincide only in that they refer to the same objects.” (van der Veer & Valsiner, 1991, p. 267; my emphasis)

Vygotsky’s knowledge of and interests concerning literary currents were also directed at Russian futurism, such as the work of the poet Velimir Khlebnikov (for traces of this interest in Khlebnikov in particular, see for example Vygotsky, 1987, p. 282; Vygotsky, 2018, p. 244, 260, 261, 274, 285, 301). One creation of the Russian futurists was the “zaum-language.” Z*aum* literally means “beyond mind,” but is more often translated as “transrational.” Métraux (2006, pp. 179–181) recalls the zaumniks’ deconstruction of speech and words to the point of a radical decomposition of everyday forms of understanding into letters and syllables. He suggests that Vygotsky might have had these and other Russian/Soviet aesthetic experiments in mind when designing psychological research such as the aforementioned experiments on concept formation. He does not provide a “hard proof” for his hypothesis, but the idea that the method of double stimulation might (also) have been generated vis-à-vis transrational poetry is attractive and holds some plausibility. In contrast to the futurists, but perhaps inspired by them, Vygotsky would, in a way, have proceeded the other way round: by offering “bat,” “dek,” “roc,” or “mup” to the children, he would have taken “zaum-words” and would have been able to observe their “charging” with sense and meaning. In doing so, he would have acted similarly to the poet and self-styled “language engineer” (quoted by Vygotsky, 2018, p. 261) Khlebnikov, who claimed a “science of word formation” was lacking (quotation of Khlebnikov by the editors of the Notebooks cf. Vygotsky, 2018, p. 268, no. 46). Indeed, the futurist poet himself even “made many experiments dealing with the semantization of individual phonemes for his poem ‘Zangezi,’ and numerous practical and theoretical tests.” (Ivanov, 1971, p. 276)

## The Semic Method

In his *Notebooks*, Vygotsky acknowledged the merits of the method of double stimulation but also criticized it for not taking meaning and sense seriously enough. Instead, he advocated the semic method, which he sometimes also called the semantic, semasiological or significative method (Vygotsky, 2018, pp. 291–309). Vygotsky advocated this method as the center of his psychological approach as sense and meaning would be constitutive of consciousness. Yet it seems more appropriate to say that sense and meaning would be constitutive for *mind in action* as this avoids giving the erroneous idea that Vygotsky wanted a solipsistic closure of mind (p. 405). For a while, Vygotsky may also have referred to *persons in action* because he seems to have had certain sympathies for Georges Politzer’s concrete psychology (p. 140 and 150 seq.; see also Kölbl, 2010, p. 153 seq.). (Incidentally, drama is a central category in Politzer’s approach).

In trying to understand the role of sense and meaning, one must emphasize that Vygotsky the semiotician cannot be separated from Vygotsky the systemic thinker (Kölbl & Métraux, 2021). What does this mean? Instead of studying the developmental path of psychic functions independently from one another, Vygotsky advocated analyzing them in their systemic interrelatedness. As soon as word and meaning come into play, the development of any psychic function alters radically. Vygotsky claimed that the task of the semic method, in contrast to the method of double stimulation, was “to study the movement of the sign itself” and that meaning was “the highest problem of the sign operation” (Vygotsky, 2018, p. 300 and 301). Moreover, “there is no psychological system without meaning” (ibid.).

The outline of a – if you will – psychological hermeneutics is particularly visible in the last chapter of *Thinking and Speech* (Vygotsky, 1987, pp. 243–285) which was partly anticipated in the *Notebooks* (see Vygotsky, 2018, p. 251) but cannot be captured in a few sentences (for more detail, cf. van der Veer & Valsiner, 1991, pp. 360–372). The following quote, however, may provide an idea. In turning to Frédéric Paulhan, Vygotsky claims that “meaning” is fixed whereas “the word’s sense is complex, fluid, and constantly changing. To some extent, it is unique for each consciousness and for a single consciousness in varied circumstances. In this respect, the word’s sense is inexhaustible. […] Ultimately, the word’s real sense is determined by everything in consciousness which is related to what the word expresses.” (Vygotsky, 1987, p. 276)

What kind of empirical studies would the semic method be appropriate for? I would argue that detailed biographical reconstructions definitely belong to those kind of studies, provided they take the wealth of the single case seriously and analyze thoroughly the complex web of sense and meaning constituting the multifaceted person-world-relations. In this context, an entry in the *Notebooks* catches my eye: “Shereshevskij must be polished as a diamond; a bright precious one that will cut through the structure of our problems and solve them like a diamond cuts glass.” (Vygotsky, 2018, p. 272) Vygotsky himself died before being able to polish this diamond but his “alter ego” Alexander Luria (Yasnitsky, 2018, p. 43 seq.) did undertake this work in his famous story of Shereshevskij the mnemonist (Luria, 1986).

## Psychology and Art

A significant part of Vygotsky’s methodologic-methodical thinking is directed towards the valorization of the single case and semiotic analyses. Art in general and literature in particular come into play in important and far from trivial ways in this endeavor. As has been repeatedly and correctly stated, it is indeed significant that Vygotsky was deeply immersed in art and literature from adolescence onwards (van der Veer & Valsiner, 1991, pp. 19–35). How then does art come into play? Or, put otherwise, what is the relationship between art and psychology? In the following, I will try to sum up the previous reflections by pointing out four thesis-like relations.

1. *Art as a subject of psychological inquiry*. Art can of course be subjected to psychological inquiry in many ways, not least in ones that do not alter psychology very much. However, Vygotsky’s way of providing a psychological analysis of art in *The Psychology of Art* is different insofar as psychology is accommodated to its subject. The creation of the objective-analytical method is one fruit of this accommodating.
2. *Art as material for illustrating and condensing psychological thinking*. Literature is used by Vygotsky to illustrate central psychological arguments or even condense important aspects of his psychological thinking, as in *Thinking and Speech* when he turns to Gumilev and Mandelstam. For a more recent example, see Jens Brockmeier (2015) on “memory, narrative, and the autobiographical process” in which the author engages (among others) with W. G. Sebald’s novel “Austerlitz” (2001) to illustrate and condense but above all to challenge the usual psychological thinking on the autobiographical process.
3. *Art as a generator of psychological methods*. The literary experiments of Russian futurism (as well as other Russian/Soviet aesthetic experiments) may have inspired the method of double stimulation. Zaumniks such as Khlebnikov deconstructed and reconstructed language, drained words of sense and meaning and refilled them, and de-semanticized and re-semanticized phonemes in quite a similar manner to Vygotsky and his colleagues’ use of (not only) the method of double stimulation.
4. *Psychology as art*. Being focused on the semiotic worlds of a mind in action, on ever-changing psychic functions in systems decisively altered by sense and meaning, not least thorough analyses of single cases are of utmost importance. In addition to the (related) objective-analytical method, Vygotsky’s “alter ego” Luria analyzed single cases through his “romantic science” in a certain way like the writer of a novel. Psychology-writing here breaks away from the conventional three-step structure of theory – method – empirical analyses and becomes an art in itself without ceasing to be science.

In concluding, I would like to return to the beginning of this paper. Moreover, I would like to do this twice, and thereby at the same moment apologize for this slightly priest-like ending. This can perhaps, however, be pardoned in a paper about a psychologist who undoubtedly sometimes held quasi-messianic convictions and compared himself with Moses (Vygotsky, 2018, p. 497).

In the introductory lines of this paper, I quoted van der Veer and Valsiner, who themselves quoted from a letter Vygotsky wrote to Luria in 1926. As you will recall, this quotation underlined the importance Vygotsky attributed to method. It was, however, incomplete and although it need not the rest may well (also) be read as a plea for psychology as a science in touch with art. The whole sentence, as quoted from Vygotsky’s letters in Georg Rückriem’s edition, runs like this: “The primary question for me, namely the question of methodology, is for me a question of truth, that it [sic!] of scientific discovery and *imagination*.” (Vygotskij [= Vygotsky], 2009, p. 216; my emphasis)

In accordance with the editors of the *Notebooks*, moreover, I stated that *ἀκμή* translates as “peak” or “zenith.” A further look into Ancient Greek dictionaries shows that it also can signify “maturation,” “blossoming time,” “blossom,” and “in its fullest bloom.” So let us just imagine that Vygotsky aimed at a psychology eventually maturing or even coming to its fullest bloom when (also) providing analyses of meaning and sense in the mind in action, not least based on really bringing complex single cases to the fore in its scientific inquiry while also in constant dialogue with art. And instead of only imagining this, let us rather work out such a psychology in detail. Vygotsky’s acmeist psychology still has some important inspirations to offer in this respect.
